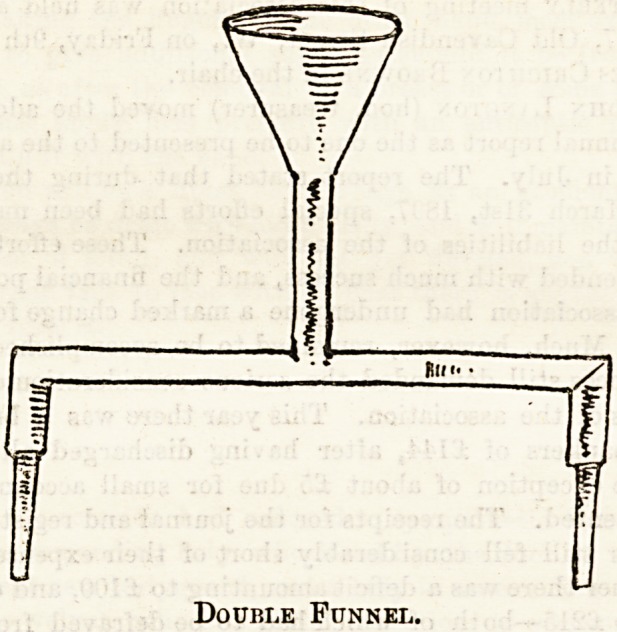# "The Hospital" Nursing Mirror

**Published:** 1897-04-17

**Authors:** 


### "The Hospital" Nursing Mirror.

Being the Nursing Section of "The Hospital."

[Contributions for this Section of "The Hospital" should be addressed to the Editor, The Hospital, 28 & 29, Southampton Street, Strand, London, W.C., and should have the word "Nursing" plainly written in left-hand top corner of the envelope.]

### News from the Nursing World.

### ROTUNDA HOSPITAL, DUBLIN.

On Saturday afternoon, April 9th, at the Rotunda Hospital, Dublin, Miss Sarah Hampson, the late superintendent, was presented with a handsome memorial of plate by the nurses, past and present, and the medical staff. Ninety-four nurses in Ireland and elsewhere signed their names to an address, which was handsomely bound in book form in crimson morocco leather. Sir Percy Grace was in the chair, and Dr. James Little, visiting physician to the hospital, formally presented the testimonial to Miss Hampson on behalf of her former pupils and staff, and spoke warmly of Miss Hampson's good work during her six years' superintendentship, especially referring to the great improvement which she had effected in the housing and domestic comfort of the nurses. The testimonial consisted of a silver tea and coffee service from Miss Ramsden, the present superintendent, and her nurses, Dr. Purefoy, master of the Rotunda, and his house staff; and a handsome circular salver from Dr. Smyly, the ex-master, and Mrs. Smyly, as a recognition of the able assistance which Miss Hampson had rendered. Lady Grace, Lady Findlater, Mrs. Little, and Mrs. Charles Martin were among the ladies present, and the nurses afterwards entertained their guests to tea in the quaint old board-room of the hospital,

### CHARITY AT HOME OR ABROAD.

A correspondent to the *Halifax Courier*, writing to support a proposal to form a nursing institution, gives an account of an effort he once made to induce the "Christian Endeavour Society" to help towards the establishment of a district nurse in a poor parish where such succour was sorely needed. "The society," he says, "finding it to be rather a heavy undertaking, very modestly placed it aside to await a more convenient season, and resolved to support two African boys on the Congo!" Yet charity is said "to begin at home." The writer concludes by urging Churches, Bands of Hope, &c., to lend their aid towards the present movement, advice which it must be hoped will be followed.

### CANON AINGER AND THE BRISTOL ROYAL INFIRMARY NURSES.

During March Canon Ainger gave two readings to the nursing staff of the Bristol Royal Infirmary. Needless to say his kindness was much enjoyed, and the readings, consisting of selections from Tennyson, Dickens, and Anstey, afforded a great treat to his appreciative audience.

### THE EDITOR OF "THE HOSPITAL."

We have received some very kind letters from nurses expressing regret that the Editor of The Hospital has been obliged to take a long holiday, owing to ill-health. We beg to thank our kind readers on the Editor's behalf, who will, we feel sure, be gratified at their kindly exhibition of interest; and we are glad to be able to say that he has already benefited by his travels and absence from work.

### THE ROYAL NATIONAL PENSION FUND FOR NURSES.

The Secretary of the Pension Fund requests us to state that the office of the fund will be closed on Saturday next, as well as on Good Friday and Easter Monday. We feel sure that nurses will be glad to hear that the Chairman has granted this little respite from work, which has been heavier than it has ever been during the past quarter, and has tried the willing staff very much.

### NORTHAMPTON COUNTY ASYLUM, BERRYWOOD.

The examinations for nursing certificates at the Berrywood Asylum have just concluded with the following results: For the examination at the end of the first year's training (first aid, &c.), fifteen candidates entered and ten passed; for the examination at the end of the second year's training (medical nursing), four entered and two passed; while for the examination at the end of the third year's training (mental nursing), the following nurses entered and all passed: Beatrice Earl (medallist), Alice Earl, May Wilson, and May Brigstock. The successful candidates will receive silver badges and certificates.

### NURSING IN CEYLON.

The work of the Ceylon Nurses' Association has steadily increased since it was first started in 1894. Up to the present time the duties which would ordinarily fall to the share of the matron or superintendent of the Home have been carried out by the Hon. Secretary, Mrs. Pole Carew. Now, however, that the new private wards for paying patients have been completed it has been found necessary to secure the services of a matron, and this office has been accepted by Miss Shankland, whose appointment will be found noticed in another column.

### NURSING AT SWANSEA WORKHOUSE.

The Guardians of the Swansea Board of Guardians have appointed a committee to "take into consideration the entire question of nursing in the workhouse," a resolution arrived at none too soon, seeing the appalling revelations made at a recent meeting of the so-called "nursing" at this institution. The question arose in consequence of the appointment of "a young tinplater" as "assistant nurse," to whom it was proposed instruction should be given in his duties by Mr. John Richards, the porter at the workhouse and late head nurse. The Rev. Father Phillipson said that the "Swansea Board stood unique in this matter, inasmuch as there was practically no trained nurse in the entire establishment, no trained nurse for the 500 or 600 inmates. In the hospital there were a lot of sick, and two or three were dying every week, and that ward was in the charge mainly of an untrained nurse." He thought the matter was most important, and he should hail the day when the Board would appoint at least one properly certificated nurse in the workhouse. It is no matter for surprise that "two or three" of the wretched patients in this forsaken place "die every week," but only matter for wonder that the death-rate is not considerably higher. But then, of course, they are "only paupers who nobody owns"!

### NURSES' HOME FOR GUY'S HOSPITAL.

IT is proposed to erect the new Nurses' Home for Guy's Hospital on a site at the junction of Great Maze Pond and St. Thomas's Street, the home being connected with the hospital by a subway under Great Maze Pond. The St. Olave's Board of Works, on being approached for permission, adopted a recommendation approving of the scheme. One member of the Board said at the meeting, when the plans came up for discussion, that he hoped "every facility would be afforded the hospital authorities. . . . A nurses' home was about to be erected, and would be for the benefit of St. Olave's and South London generally."

### STEEVEN'S HOSPITAL, DUBLIN.

A NURSING HOME is to be erected for the staff of Steeven's Hospital, Dublin, in commemoration of the present year, and the foundation-stone is to be laid on the anniversary of the Queen's accession. Steeven's Hospital has many claims upon public support; it is the oldest hospital in Dublin and it is largely used for the benefit of the employés at breweries, distilleries, and railway works in the western quarter of Dublin; it has also a special ward for sick men of the Constabulary. A home for the proper and adequate accommodation of the nursing staff is much needed, and the project is receiving encouraging support.

### NURSES v. NEIGHBOURS.

THE oddest argument against district nurses is reported to have come from a clergyman at a recent meeting held at Heanor to consider schemes for the commemoration of the Queen's reign. A scheme to provide two district nurses for the parish was the proposal which met with the most approval, but the Rev. George Avis advocated a recreation ground, stating that "he had a strong feeling against a nurse scheme because to his mind it would greatly interfere with neighbourly feeling in times of sickness." One would like to ask Mr. Avis if in the event of serious illness he would feel happy at entrusting the care of his nearest and dearest to the nursing of a "neighbour"!

### BRISTOL DISTRICT NURSES.

THE annual meeting of the Bristol District Nurses' Society was held last week, presided over by Mr. J. Storrs Fry. The report showed that the 16 district nurses had been in constant employment during the year, and that their visits were much appreciated. There was unfortunately a deficit of £48 on the account. The Chairman expressed the hope that a society doing such excellent work would not suffer from want of funds; he could speak from his own knowledge of the beneficial effects of the nurses' visits to the poor, and paid a tribute to the "skill and kindness" of Miss Lloyd, the Lady Superintendent. A lady guardian who was present spoke of the appreciation which the guardians felt for the work of the nurses, and instanced the fact that the nurses had paid 7,000 visits in the Barton Regis District last year. She believed the Guardians would raise their subscription.

### KILFINICHEN, MULL.

NURSE MARION MACDONALD, district nurse at Kilfinichen, Mull, is giving up her work to be married. A farewell ceremony was got up in her honour in the Creich Schoolroom, when a handsome gold watch and chain and a purse of money were presented to her. The presents were accompanied by many warm good wishes for "Nurse's" happiness. The watch is engraved with this inscription: "Presented by the people of the parish of Kilfinichen, Mull, to Marion MacDonald, Queen's Nurse, on the occasion of her marriage, and in appreciation of her services as district nurse. March, 1897."

### EDINBURGH NURSES OFF DUTY.

NURSES from all the hospitals in Edinburgh were recently invited by the Corporation of the City to an entertainment at the Freemasons' Hall. Lord Provost M'Donald and Bailie Pollard, convener of the Public Health Committee, received the guests, some three hundred and fifty in number, amongst them being a deputation from the Belvidere Hospital, Glasgow. Sir Henry and the Misses Littlejohn, Professor Chiene, Dr. Joseph Bell, Surgeon-General Lithgow, and many other guests were present. The nurses came in uniform.

### NORTH LONDON NURSING ASSOCIATION.

A SALE of work is to be held at the North London Nursing Association Home, Holloway Road, on May 19th and the two following days, for which contributions in money and kind are earnestly asked. The association does an excellent work in North London, and deserves to be well supported. Anyone wishing to aid its cause by helping with the forthcoming sale should communicate with the Misses Hollway, 6, Highbury Grange, N.

### SHORT ITEMS.

MRS. COSTER, who has filled the office of Superintendent of Nurses and Matron at St. George's Hospital for twenty years, has resigned her appointment.—At Hadleigh it has been decided to celebrate "the Great Jubilee" by raising a "Nurses' Fund."—The Grimsby Nursing Association has been affiliated to the Queen's Jubilee Institute during the past year.—Nurse Underwood, who asked through our "Wants and Workers" column for a bath-chair for the crippled patients in her parish, writes to tell us that a lady has sent her "a very nice one." We are sincerely glad that her appeal has met with this success.—Elizabeth Legge, "midwifery nurse" at St. George's Workhouse, Southwark, recently committed suicide at the workhouse by taking carbolic acid. It appeared from evidence at the inquest that she had been in a depressed and despondent frame of mind for some time. The jury returned a verdict of "Suicide whilst temporarily insane."—The Lady Mayoress has granted the use of the Mansion House for an entertainment to be given through the "Children's Salon" in aid of the Commemoration Fund for the Queen's Jubilee Nurses.—A donation of £50 has been sent anonymously to the Glasgow Sick Poor and Private Nursing Association. The association is affiliated with the Q.V.J.I.—The committee of the Gateshead Children's Hospital have decided to found a cot in commemoration of the Queen's reign, to be called "The Queen's Diamond Jubilee Cot."—A meeting of members of the Birmingham and Midland Pharmaceutical Students' Association for Women took place at the Women's Hospital, Upper Priory, Birmingham, on Friday, April 9th. The subjects for discussion were "The Education of Dispensers" and "The Desirability of Extending the Association."

### Lectures to Surgical Nurses.

By H. A. LATIMER, M.D. (Dunelm), M.R.C.S. of Eng., L.S.A. of London, Consulting Surgeon, Swansea Hospital; President of the Swansea Medical Society; Lecturer and Examiner of the St. John Ambulance Association, &c.

### V.—HÆMORRHAGE; MEANS TO BE ADOPTED FOR CONTROLLING AND ARRESTING BLEEDING.

In my last lecture I told you that I would next instruct you in the measures to be adopted for the control of bleeding. Starting with a surgical case:—

If it be a limb which has to be amputated, or which has to be operated upon, whilst the patient is under chloroform, it is held up in such a way that it is well nigh drained of blood, and, when it is judged that that result has been attained, an india-rubber tube, having a hook at one end and an eye at the other, after having been put on the stretch, is wound two or three times around the limb, above the point where it is intended to operate, and is there secured by hitching the hook and eye together. The operation is now performed. After the removal of the diseased part the cut vessels are looked for, found, seized with appropriate forceps, and tied with one or more of the antiseptic ligatures which are now in vogue. Now the band (called a tourniquet) is removed. If the arteries have been securely tied they will be seen beating, but not bleeding. Still a good deal of blood may be flowing from the general surface of the wound. This may be venous. Very hot water impregnated with some antiseptic is now poured over the wound, whilst the limb is held up, and if the great vessels have been secured, as has been thought, the contraction in the smaller ones induced by this heat will be sufficient to stop their bleeding. It may be, though, that the patient is so faint or collapsed from the operation itself, from his previous condition, or from the action of the anæsthetic he has taken, that vessels may not bleed now, but may do so later on; a clot which has sealed a vessel may become dislodged, and there may be a sudden furious gush of blood; then we are said to have recurring hæmorrhage. I shall return to these later bleedings when I come to tell you of your duties as nurses.

In cases of hæmorrhage not caused by the surgeon, his action has to be speedy and energetic. One of the first things he has to do is to bare the bleeding part, and see actually from whence the blood issues. Should it come from an artery, he will put his thumb on a part of the vessel higher than the bleeding point, and, if he has rightly gauged the case, the pressure which he will then make will stop the bleeding below. This is called stopping bleeding by making digital pressure. To do it you must know a sufficiency of anatomy to tell you how the main vessels run. The main artery of the upper extremity emerges from under the collar-bone into the arm-pit, and from thence, under the name of the brachial artery, it makes its way down the inner side of the arm to the middle of the bend of the elbow, taking a line which is well indicated by the seam in a coat-sleeve, as it does so; at the elbow it divides into two main arteries, called the radial and ulnar, which run down the inner and outer sides of the fore-arm to the wrist, from whence they pass into the hand. The same arrangement applies to the arteries of the lower limbs, for the thigh has one large artery, the femoral, which runs from a point mid-way in the groin to the inner side of the limb, just above the knee, and then makes its way to the hollow behind that joint, and the leg has two large arteries, the anterior and posterior tibial, which run down among the muscles to supply the foot. Notice, now, how when I press this brachial artery the pulse ceases to beat at the wrist. You can see how easy it is to stop the flow from a single vessel in this way. Then the surgeon proceeds to tie the vessel which is causing this trouble, for he never rests content with a bleeding artery until he has secured it in this way.

An accident which is exceedingly apt to occur is that of the rupture of a varicose or tortuous vein in the leg. Elderly people who have suffered from these diseased veins are particularly liable to this danger, as the skin overlying the faulty vessels gets thin and stretched, and is apt quite suddenly to give way. Then dark blood is felt and seen to be trickling down the leg. With knowledge such cases are easily dealt with. The patient is immediately laid on a couch or bed, the leg is raised and a pad is placed on the bleeding spot, and retained there by a roller bandage which has been applied from the toes upwards, and the bleeding ceases. Elevation of the limb is of prime importance in all hæmorrhages, because by doing this you prevent the blood going easily along the arteries; and if these vessels are empty it must stand to reason that the veins must be empty too. Blood is like any other fluid, and will run with the greatest ease along a level plain or down hill. Our limbs are constructed to favour this. The head and neck are the only parts where blood has to go up-hill, and it is enabled to do so here by the fact that it is circulating in large vessels which are within easy influence of the pumping action of the heart, and which are not of any considerable length.

When the surgeon is operating for the removal of tumours in situations where bleeding cannot be controlled beforehand, severed vessels are seized at once with forceps and tied, or fingers are placed immediately on bleeding points, and pressure is kept up until the forceps are available.

Your duties may be regarded here from the point of view of your care of a bleeding person, or of one who has bled and is liable to bleed again. The first event might happen if you were in charge of a cottage hospital; the second, while watching a patient who had been operated upon. In both cases, if the bleeding should be from a limb, remember what I have said about having the same raised; do this, and continue to do it until the surgeon comes to relieve you of your anxieties. Should the bleeding not be excessive do not concern yourselves much as to its cause, leaving that matter to be decided upon by the doctor; content yourself with applying an antiseptic pad of some sort to the bleeding part, and maintain it firmly in place with a roller bandage. Should the bleeding be very severe, and especially if an artery is its source, apply digital pressure, or encircle the limb with an elastic tourniquet. You can very effectually stop bleeding from arteries in the leg, foot, and in the arm, forearm, and hand, by placing pads in the hollow behind the knee, in the arm-pit, or at the bend of the elbow joint, and then doubling the appropriate limb on the pad, and applying a bandage to keep the bent limbs together. Any bleeding vessel in the neck (as in the case of cut-throat) must be dealt with by firm pressure with the thumbs over an antiseptic pad placed on the bleeding spot; you must not leave such a case until relieved by the medical man, and must not trust to a bandage to keep the pad in place. Wounded vessels on the head and face are easily dealt with, as they run over bones which afford good points for counter pressure; these you may pad and bandage with safety.

It is not often, however, that you will have to do these things, as you will mainly be employed in looking after the patients who have undergone operations, or who have been attended by surgeons already. In such cases bleeding will already have been arrested, and the patient will have been placed in bed under your charge.

You will now have to watch carefully lest dressings should become displaced during the restlessness which is apt to accompany "reaction," restraining movements if they seem likely to do harm in any way. Should any blood show itself outside the dressings you will be guided in your action by its amount, and the rapidity with which it appears. If it should be mere oozing you may content yourself with applying some greater pressure over the dressings by some antiseptic wools and firmer bandaging without disturbing them, telling the surgeon what you have done on his next visit. But if much blood should come and the dressings should be quickly saturated with it, send at once for the surgeon in attendance and deal with the case on its merits; should it be after an amputation, raise the stump and encircle it well above the vicinity of the wound with your hands, pressing the fingers of one hand on the main artery, and making the fingers of the other hand meet over their fellow to give additional support; should it be from some wound, as after the removal of a breast or of some tumour, you must apply firm pressure with an antiseptic pad and maintain it with the hand.

If you will look upon hæmorrhage as an accident which can always be dealt with most easily, and will maintain your presence of mind, and will not get flurried, you will not find, when face to face with it, that there is anything to be feared from it.

### À Crete.

On Thursday morning Mrs. Ormiston Chant and six nurses left England *en route* for Crete viâ Athens. Mrs. Chant, Lady Henry Somerset, and Mrs. Bedford Fenwick have, we understand, been the organisers of this expedition. For funds, we understand that Mrs. Chant asked for at least £900 to be subscribed by the public to carry out the scheme, and it is stated that an appeal for "the Cretan Wounded Nursing Fund" on the part of the *Daily Chronicle* has resulted in some £400; while Lady Henry Somerset collected donations from her "Women's Society" and the "Women of America" of £100 each. A collection on behalf of the fund was made at Pembroke Chapel, Liverpool, last week, the congregation passing a resolution heartily congratulating "Mrs. Ormiston Chant and her heroic colleagues upon their determination to run the blockade of Crete and to offer their services to the sick and wounded." The *Times* says that "Mrs. Chant told a correspondent before leaving that war, or no war, they hoped to find some work of kindness and mercy so that their journey would not be an idle one." The following amusing account of the departure of the party from Charing Cross we reprint from the *Daily Graphic* of the 9th inst.:—

Cretan Pilgrims.—Departure from Charing Cross.

At a quarter to eleven yesterday morning the two policemen in charge of the gates of the Continental departure platform nearly gave up their post in despair. Excited ladies were appealing in crowds to be allowed to go on to the platform. "Policeman," cried one of them, "can you tell me if dear Mrs. Ormiston Chant has yet arrived?" "Oh," said the policeman, "here's another of 'em! Yes, ma'am," he continued, respectfully, "she has." "Then," pursued the lady, "will you permit me to pass?" "I want to go on, too," exclaimed another lady. "Policeman," cried a third, "I must go on! I'm going to see a friend off to Crete." "So am I." "And I." "And I," chorused the others. "Here, Alfred," remarked the stout policeman to his colleague, "let 'em all on!"—and in the rush which followed the representative of the *Daily Graphic* contrived to struggle on to the platform. When he had recovered his hat and replaced his spectacles, an interesting sight met his eyes. In the door of a saloon carriage stood a stout little lady in a nurse's uniform of grey dashed with red. She had what appeared to be a cartridge-belt round her waist, and she carried a bunch of pure white roses in her hand. Somewhere mingled with the background were other nurses in blue dresses with a scarlet Maltese cross embroidered upon them, and planted thickly about the platform was a dense crowd of ladies saying good-bye. There were soulful ladies anxious that their names should be given to the reporters, there were ladies in spectacles and sensible boots, there were vivacious ladies, there were ladies whom the occasion had reduced to sympathetic tears, there was a commanding lady who stood by the door of the compartment as a bodyguard, and a lady in an exciting costume of blue velvet, who was giving directions to everyone; and there were a few murky curates. On all these Mrs. Ormiston Chant was smiling with a feverish geniality, and the *Daily Graphic* representative, momentarily anticipating a speech, strove hard to get at his pencil in the crush. It was not to be, however, and even the information he was successful in obtaining was purely fragmentary. "Are you going?" asked one nurse of another, whose umbrella was within a sixteenth of an inch of the *Daily Graphic* eye. "No," replied the other, "but my sister is one of the nurses at the Home." "Is she going to Crete?" pursued the other, "How nice!" "No, she's not exactly going to Crete," said the first, "but she's going as far as Dover with them. The railway company has given them free passes." "How noble of them," remarked the lady with the umbrella; "and is dear Mrs. Chant going to Crete?" "I don't think so," responded the lady with the sister. "I think she's going to Athens to speak!" "How like her," agreed the umbrella, "*dear* Mrs. Chant!" But even when dear Mrs. Chant is leaving us, leave-taking cannot last for ever. The inevitable guard came to take the tickets, and Mrs. Ormiston Chant unflinchingly opened her cartridge pouch and withdrew the vouchers for Crete (six nurses), and Athens (one, Mrs. Ormiston Chant). The door was closed, Mrs. Chant once more kissed everybody within reach, and the train began to move. At the very last moment a belated lady rushed up unfurling a Cretan flag of the type made familiar to Londoners by the news carts of a well-known evening paper. At this demonstration one of the curates waved his hat and cheered; the ladies on the platform joined him in a shrill "Hooray!" and the train steamed out with Mrs. Chant smiling to the last. "How very sweet," said the lady with the umbrella, as the crowd turned away. "Isn't it?" said the other; "and do you know, dear, they've all been photographed, and their portraits are going to appear somewhere!" "*Dear* things!" observed the umbrella.

### Where to Go.

Galleries of the Royal Institute of Painters in Water Colours, Piccadilly, W.—A ball in aid of the Ospedale Italiano (Queen Square, W.C.) will take place at the above galleries on May 3rd, under distinguished patronage. Tickets and all information may be obtained from the Duchess San Germano Di Calabritto, 10, Emperor's Gate, S.W.; Lady Cunynghame, 134, Cromwell Road, S.W.; or Mrs. L. Le Trobe Bateman, 18, Walton Place, S.W.

### Wants and Workers.

Nurse Peters, 109, East Street, South Molton, Devon, has about 30s. worth of diabetic food, buscuits, chocolate, flour, &c., to give away, and will be glad to hear of someone or some institution to whom it would be of use.

### Royal British Nurses' Association.

A QUARTERLY meeting of this association was held at the offices, 17, Old Cavendish Street, W., on Friday, 9th inst., Sir JAMES CRICHTON BROWNE in the chair.

Mr. JOHN LANGTON (hon. treasurer) moved the adoption of his annual report as the one to be presented to the annual meeting in July. The report stated that during the year ended March 31st, 1897, special efforts had been made to reduce the liabilities of the association. These efforts had been attended with much success, and the financial position of the association had undergone a marked change for the better. Much, however, remained to be accomplished, for the finances still demanded the serious consideration of the members of the association. This year there was a balance at the bankers of £144, after having discharged all debts with the exception of about £5 due for small accounts not yet presented. The receipts for the journal and registration accounts still fell considerably short of their expenses; on the former there was a deficit amounting to £100, and on the latter to £215—both of which had to be defrayed from the general fund account. The amount advanced by the general account to the Registration Board amounted to £1,354 12s. 6d. In conclusion the report drew attention to the heavy legal expenses, which amounted to £206, incurred during the year, and also suggested that this, the sixtieth anniversary of the Queen's reign, is a fitting opportunity for the association to raise a fund to replace the invested capital which was expended in obtaining their Royal Charter in 1893.

Mr. F. J. GANT seconded, and the report was adopted.

Mr. E. A. FARDON (hon. medical secretary) presented the report of the Executive Committee, and proposed that it be accepted by the Council. In that report the Executive Committee referred to an action which had been brought against Miss de Pledge to recover damages for an alleged libel said to have been published in the *Nurses' Journal* of August, 1896, and stated that they had referred the matter to the hon. treasurer and the hon. secretaries as a sub-committee, with power to defend the action on behalf of the association.

Dr. ALDERSON seconded, and the report was adopted, three voting against the motion.

The nominations of members of the General Council for 1897-8 were passed by the meeting.

It was decided, on the motion of Mr. E. A. FARDON, that an address of congratulation on her glorious reign be presented to the Queen, the details being left to the President and the Executive Committee to arrange.

The suggestion as to raising a fund to replace capital expended mentioned above was also referred to the Executive Committee.

A vote of thanks to the chairman concluded the business.

### Novelties for Nurses.

### NOVELTIES AT MESSRS. GARROULD'S.

MESSRS. GARROULD are just now showing some new designs in nurses' cloaks. Most of them are smart and eminently becoming, but we cannot help deprecating from a professional point of view the somewhat increasing tendency towards the fashions of the day in nurses' uniform. A nurse's uniform, like a soldier's, should never vary. There should be a regulation cut about it suggestive of business rather than pleasure. This idea we find most fully carried out in the always to be admired "Angelus" cloak. Messrs. Garrould are to be congratulated on the elegance and style of this particular shape. It sits well to the figure and gives an air of distinction to the most ordinary wearer. It can be had in either fine all-wool cravenetted cashmere cloth thoroughly waterproofed for 21s., or in all-wool serge for winter wear at 23s. 6d. The shape we admire next to this is the "Ellesmere," which is conveniently fitted with a three-quarter cape. In fine cashmere the price quoted for it is only 18s. 9d., and for about 4s. or 5s. extra it can be had in all-wool serge. This cloak we recommend especially district nurses; it is admirably adapted to the more varied conditions of their existence, and would look well on a bicycle. The "Glen Mary" is a charmingly shaped cloak of the time-honoured circular pattern, always so attractive and becoming. We should prefer it, however, without the frill round the neck, which, in our opinion, detracts from the elegant simplicity which is its distinguishing feature. In cravenetted cashmere the price is 27s. 6d., which is not expensive for the class of article. The "Hebena" and the "St. Katherine" are both very pretty cloaks, but more adapted to the lay than the professional wearer; to the former we have every confidence in recommending them as both stylish and fashionable, and we predict for them a large sale. Nurses' gowns also receive the attention of this enterprising firm, who have one of the best selections in the metropolis of materials suitable for institution wear. First-class fitters reside on the premises, and customers can be attended to at the time of purchase without any vexatious delay. Messrs. Garrould's special Halifax linens are simply lovely. All the newest shades are produced in them at marvellously low prices. The great thing to ask for is the new 40-inch cotton cloth "Garrouldetta," which is quite a novelty, very serviceable and pretty, and only 1s. 0½d. per yard. We specially desire to draw the attention of our readers to an all-wool washing Beige, "The Harrisburg;" it is an admirable fabric, and only costs 12s. for a dress length. The "Sister Dora Cap," so justly popular, can be had at a shilling, and a pretty shell-shaped one, "Sister Mabel," for 1s. 6d. The sick-room slipper, however, we must call special attention to. The soles are of twine, very durable, and quite noiseless, and the upper part of felt, warmly lined throughout with scarlet flannel. A new walking shoe, bearing the appropriate soubriquet, "Ease at Last," will supply a long-felt want of something soft and easy to the foot, while presenting a neat appearance on the outside. Nurses' wallets and chatelaines are kept in all varieties. In fact, there is no department affecting a nurse's interests that has not been carefully planned and fully equipped by Messrs. Garrould.

### Appointments.

VICTORIA HOSPITAL, FOLKESTONE.—Miss Martha Cook has been appointed from amongst a number of candidates to the Matronship of this hospital. Miss Cook was trained in the Nightingale Fund Training School, St. Thomas's Hospital, subsequently being appointed day and then night staff nurse at St. Thomas's. For the past two years she has held the post of sister at the Victoria Hospital, Folkestone. We heartily congratulate Miss Cook on her promotion.

ACCRINGTON COTTAGE HOSPITAL.—Miss M. E. Burgess has been appointed Nurse-Matron at this hospital. Miss Burgess was trained at the Adelaide Hospital, Dublin (4½ years), and was for three months at the General Hospital, Nottingham. Her subsequent appointments have been as charge nurse at the Fountain Fever Hospital, as sister-in-charge of Home Hospital, Dublin, and as night superintendent at the Blackburn Infirmary.

CEYLON NURSES' ASSOCIATION, HALTON, CEYLON.—Miss Shankland, who has been for nearly three years working for this association, has been offered, and has accepted, the new appointment of Matron to the association. Miss Shankland was trained at Crumpsall Infirmary, Manchester, and the Leeds Trained Nurses' Institution, Leeds. Since then she has been engaged in private nursing.

DENILIQUIN HOSPITAL, NEW SOUTH WALES. — Sister Blanche Bucknell has been appointed to the Matronship of this hospital. Sister Bucknell was trained at the Prince Alfred Hospital, Sydney, where she has held the post of sister to the present time. There were many applicants for the vacant appointment, and Sister Bucknell is to be congratulated on her success.

### Nurses' Inventions.

NURSES are generally ingenious people, and many clever little contrivances are evolved by them for the comfort of their patients and the saving of time and trouble in the sick room or hospital ward.

The accompanying illustrations show a fire-guard invented and patented by Mrs. F. Cunningham Woods, "an old nurse," the idea of which arose in her desire to prevent falling cinders from awaking her patients. It will be seen that it has hinged wings or side-pieces, which encircle the fire, and an adjustable shelf of wire, upon which the coals fall with little sound. In the latest ones made, a little shelf

FRONT (OPEN).

BACK (CLOSED).

"WOODGATE" FIRE GUARD.

has been added on the outside of the guard, on which anything to be kept warm could be placed. This guard keeps the hearth tidy and the fire perfectly safe; when not in use it will fold flat and pack into a small space for travelling. The "Woodgate" guard can be made in brass, price 11s., or in ordinary iron wire for 6s. 6d. It may be ordered through any ironmonger, or from the maker, Mr. J. Clark, 47, High Street, Bloomsbury, W.C.

A "Hospital Sister" sends us illustrations of two special

STAND FOR FEEDING BOTTLE.

contrivances of her own which she has found of use in her ward. The first is a stand for holding a baby's bottle, and the other a hot water tin-filler. The bottle-holder is arranged in order to permit the busy nurse to see to the feeding of several babies at once, placing the stand on the cot, and regulating the position of the bottle by means of the graduated support shown in the drawing. It is not stated what is to regulate the baby's hands.

DOUBLE FUNNEL.

The second drawing shows a funnel specially made with the idea of filling two hot-water tins at once, a plan which the nurses find very convenient.

### Sketches of Hospital Life.

### THE RULING PASSION.

The ruling passion, be it what it will,
The ruling passion conquers reason still.—*Pope.*

A FEARFUL railroad collision had taken place. From early morning the ambulances had been discharging their ghastly loads at the hospital door. The corridors were filled with heartbroken men and women, waiting for news of the dying, or, perhaps, even then, the dead. Within the wards, it seemed that chaos had come; beds were hastily improvised, the small hospital being quite inadequate to this sudden influx. The staff of nurses were taxed to the utmost, the heartrending and sickening sights proving too much for some of the new probationers, who came to the conclusion that nursing was not, after all, their vocation. An Irish boy, one of the railroad employés, was placed in a small room; he was doomed from the first; the doctors thought he could not possibly survive the night. He had borne his sufferings with the heroism and patience of his race, always thanking the nurse for the little she could do. When evening came, he still lingered, and when his mother appeared, to remain beside her dying son, her offer to take charge of him was gratefully accepted by the nurse. Directing her to give a small amount of brandy every half-hour, and placing a bell within her reach, she left mother and son together, intending to return very shortly. The hours flew along, the distracted nurse hardly found time to breathe, expected help did not arrive, and some lives must be saved. Towards the dawn of a winter's day she remembered, with a pang of conscience, the dying patient, and, wondering why the mother had not called her, opened the door. One glance at the bed told her that the boy was resting easily in the "long sleep of death." The time he had died was unknown. The odour of brandy filled the room, the bottle stood empty on the table, and on the floor in a drunken stupor lay the wretched mother.

### Minor Appointments.

BOSTON UNION.—Miss Maude E. Hill has been appointed Head Nurse of this Union. Miss Hill was trained at Fir Vale Infirmary, Sheffield, where she remained for six years, leaving to take up her new appointment.

### Everybody's Opinion.

[Correspondence on all subjects is invited, but we cannot in any way be responsible for the opinions expressed by our correspondents. No communication can be entertained if the name and address of the correspondent is not given, or unless one side of the paper only is written on.]

### ING'S HOUSE NURSES' CO-OPERATION AND THE LONDON ASSOCIATION OF NURSES.

The Lady Superintendent of the London Association of Nurses, 123, New Bond Street, writes: Should you decide to make any comment on the action against the proprietress of the Ing's House Nursing Co-operation, of 81, New Bond Street, heard at Lambeth County Court on Wednesday last, would you kindly mention that it has no connection with this association, as we find that some confusion about the two places exists in the public mind.

### NURSES' EARNINGS.

Some "Jubilee Nurses" write: We, as a body of the Queen's nurses in the City, endorse the statement of the nurse who asks "Why nurses should be paid such small salaries?" We have long felt that a higher standard of salaries is needful to nurses in general, especially district nurses, considering the many difficulties we have to cope with and the depression of the work, which none can understand except district nurses themselves. We are pleased with the advancement of district nursing in connection with the Queen's Jubilee, but certainly think the nurses' salaries might be increased, especially to commemorate such an event as the Diamond Jubilee, as Her Majesty appreciates our work so much. We shall be pleased to hear if other Jubilee nurses are of the same opinion.

### ASYLUM NURSES.

"One of the First Thousand" writes: I have just come across a paper called *Asylum News*, and, by it, find that a "home of rest" is contemplated for asylum workers; and not a day too soon. I have often wondered why some philanthropist's heart has not been moved to do something for this hard-working class. I was an asylum nurse myself for four years, many years ago, but found it so terribly trying to nerve and body, and met so little sympathy (I did not expect gratitude) from all concerned, that I gave it up and took to sick nursing, to which I have adhered since. But although I am not a mental nurse now, I still sympathise with my sister nurses in the asylums, and often wish I were in a position to help them to receive the due recognition their very arduous work deserves. In looking over the balance-sheet in the above paper, I find the cash in hand is under £100. With this sum I cannot see how a "home of rest" is to be established; but if every medical superintendent subscribed £1 to the subordinate members' modest shilling, it would be a substantial addition to the fund in hand. With every respect, I make this suggestion through your valuable paper, which I always find is the first in the field in taking up the cudgel on behalf of all hard-working nurses. Would it not be possible to get a small share of the fund that is being collected for the benefit of the sick nurses during this record year set apart for the benefit of mental nurses? It must be very disheartening to the asylum nurse to see how much is being done for her hospital sister, while she is still neglected. It is a case of one getting the kicks and the other the ha'pence.

### A "UNIFORM" QUESTION.

"Another District Nurse" writes: I always think that all nurses when off duty would benefit themselves and others by discarding their uniform altogether, and wearing some quiet, unobtrusive attire. They would thereby escape being specially observed and talked about, and would save themselves from having their off-duty time robbed of its recreative effects by being compelled to answer innumerable questions about their work and patients, which their uniform suggests to the minds of both strangers and friends. A sister from a London hospital once told me that she was persuaded to wear her uniform during her summer holiday, with the result that she had not been able to get through one day without questions about her training, work, &c. Off-duty time and holidays should be spent by nurses in giving both brain and body rest, and getting fresh ideas and views, which will enable them to return to their work refreshed. If a district nurse desired to be "the observed of all observers" at an evening party, there is no reason why she should not go in the uniform she wears when at work, provided she does not say or do anything unworthy of her high-calling. But a fancy uniform dress I should think fitter for a lady who wanted to represent a district nurse at a fancy ball. Any lady who objected to gay attire would look exceedingly genteel at any evening party in a navy blue silk dress, plainly made, with lace ruffles instead of uniform collar. As a hospital nurse I found my quiet durable attire did not cost more than many of my friends spent on their uniform. As a district nurse I have always found it beneficial and refreshing to doff my uniform for a few hours or during holidays, and pass unnoticed among my fellow-reatures.

### ALMSHOUSES FOR NURSES.

"A. B. M." writes: I have read with much interest the letter headed "Almshouses for Nurses." A similar scheme had been in my mind for the last five years, and I have only been waiting my opportunity to bring the matter before my professional sisters' notice. My own idea is briefly this—First, let all qualified medical and surgical nurses (not monthly nurses, as they often begin work when elderly) at once pay a yearly subscription of two guineas towards a real home for old age or when incapacitated for a time for nursing by ill-health. Second, let nurses willing to subscribe such sums band themselves into a guild, to be called the Victoria Guild for Old Age and Sickness. Third, let the money given and subscribed be delivered into the care of a private person (gentleman preferred) who will act as treasurer. Fourth, the word "hall" to be substituted for that of "almshouse," as the society will be, after the preliminary expenses of building and endowment, a self-supporting one. Fifth, the scheme to be mainly upheld by nurses, and not laid too prominently before the public; at the same time, nurses' relations and friends interested may be asked, but not urged, to contribute towards the preliminary expenses, and donations and subscriptions may be *received by the treasurer only*, by whom all amounts will be acknowledged. Sixth, printed forms regarding the contemplated enterprise to be distributed by the nurses amongst their own friends and others. Seventh, as the hall would not be required for elderly nurses for some years to come, the dwelling would be made ready to receive delicate or disengaged nurses looking out for suitable engagements at a weekly charge of 10s. In this way, a moderately-sized endowed hall might be started within the year and maintained, provided that all applicants were members of the Guild. This place might be enlarged as time passed, and the fees accumulated, and other buildings erected, including those at the seaside. Eighth, the whole affair to be managed by trained nurses, and a matron of their own choice appointed to preside over the hall. I venture on making these suggestions, and shall be glad to hear those of other nurses. The expression, "Home of Rest," &c., is used for "Friendless Girls' Homes," and is not altogether suitable; but I anticipate. As many nurses marry; as many die in the performance of their duties; and others have no need to avail themselves of a self-supporting refuge for old age—should most nurses subscribe out of the fulness of their sisterly hearts, for the good of others, if not for themselves, a substantial sum might be realised in ten years' time, before any aged person sought shelter. One thousand nurses alone, subscribing two guineas per annum, would realise the sum of £20,100 in that time. But, and there is always a "but," we must look before we leap. Such a place ought to be properly endowed at starting, or the whole affair might collapse. But surely a sufficient sum might be raised this Jubilee year to build and endow a hall containing 30 beds. Lastly, I should like to suggest that when elderly nurses (who had been regular subscribers from the first) took up residence in the hall, they should not be put to any expense save that of personal clothing, and perhaps washing of the same. For this purpose members of the Pension Fund would have ample means, and no doubt many others, who for various reasons had not been able to join that admirable society, but had nevertheless saved a little in later years. A healthy place within twenty miles or so of London, such as Ascot, would be perhaps best for the Pioneer Hall. I myself am a qualified nurse of twelve years' standing; was trained at Addenbrooke's Hospital; and am thirty-five years of age. If, sir, you would kindly allow nurses who are willing to join the Victoria Nurses' Guild (and subscribe two guineas per annum) to send in their names to you to be inserted in your valuable paper, I believe a great start in the right direction would be made.

### For Reading to the Sick.

EASTER.

CHRIST is risen from the dead, and become the first fruits of them that slept.

Christ is risen! We are risen!
Shed upon us heavenly grace,
Rain and dew and gleams of glory
From the brightness of Thy face;
So that we, with hearts in heaven,
Here on Earth may fruitful be;
And by Angel-hands be gathered,
And be ever, Lord, with Thee!
—*C. Wordsworth.*

Thou know'st He died not for Himself, nor for Himself arose;
Millions of souls were in His Heart, and thee for one He chose.
Upon the palms of His pierc'd Hands engraven was thy name,
He for thy cleansing had prepar'd His water and His flame.
Sure thou with Him art risen; and now with Him thou must go forth,
And He will lend thy sick soul health, thy strivings might and worth.
—*Keble.*

We would indeed be somewise as Thou art,
Not spring, and bud, and flower, and fade, and fall—
Not fix our intellects on some scant part
Of Nature,—but enjoy or feel it all:
We would assert the privilege of a soul,
In that it knows, to understand the Whole,
If such things are within us—God is good—
And flight is destined for the callow wing,
And the high appetite implies the food,
And souls must reach the level whence they spring!
O Life of very Life! set free our Powers,
Hasten the travail of the yearning hours.
—*Houghton.*

Now once more, Eden's door, opened stands to mortal eyes,
For Christ hath risen, and man shall rise.
—*Neale.*

**Reading.**

I am the Resurrection and the Life.

Let this be thy whole endeavour, this thy prayer, this thy desire; that thou mayest be stripped of all selfishness, and with entire simplicity follow Jesus only; mayest die to thyself, and live eternally for Me.—*Thomas à Kempis.*

That which thou sowest is not quickened unless it die. —1 *Cor.* xv. 36.

As He is risen, so now He dieth not. The widow of Nain's son, the ruler's daughter, Lazarus—all these rose again, yet they died afterwards; but Christ rising from the dead, dieth no more. If we rose as they did, that we return to this same mortal life of ours again, this very mortality of ours will be to us as the prisoner's chain he escapes away withal; by it we shall be pulled back again. We must, therefore, so rise as Christ, that our resurrection be not a returning back to the same life, but a passing over to a new. The very feast itself puts us in mind of as much—it is the Passover; not a coming back to the same land of Egypt, but a passing over to a better—the Land of Promise, whither Christ, our Passover, is passed before us, and shall in His good time give us passage after Him.—*Bishop Andrewes.*

### Notes and Queries.

*Hospitals in South Africa.*

(210) Can you tell me anything about hospitals in or near the Orange Free State. Would probationers be taken there, and for how long a term of training?—*Senga.*

You had better write direct to the various hospitals in or near the Orange Free State and ask for full particulars of the training and if probationers are wanted. There is the St. George's Cottage Hospital (Sister Louisa Fane) and Volk's Hospital, Bloemfontein; and the Jagersfontein Hospital, in the Orange Free State. In the Transvaal you might write to the Johannesburg Hospital, the hospitals at Krugersdorp and at Barberton, and Volk's Hospital, Pretoria. In Basutoland there is the Masern Hospital, where Dr. E. C. Long is the medical officer.

*Pension Fund Nurses.*

(211) When are the Pension Fund nurses likely to be presented to the Princess of Wales?—*Rob Roy.*

Apply to the Secretary of the Royal National Pension Fund for Nurses, 28, Finsbury Pavement, E.C.

*Sanitary Inspectorships.*

(212) Please tell me the address of the Sanitary Institute in London. Also should you recommend going in for postal lectures with a view to qualifying as a sanitary inspector?—*L. P.* and *Sanitary Inspector.*

The address of the Sanitary Institute is 72 and 74A, Margaret Street, W. Miss Lampert, 52, St. John's Wood Road, N.W. (a member of the institute), has carried on very successful correspondence classes for some years past for the benefit of those wishing to become sanitary inspectors, and anxious to pursue their studies without interfering unduly with other work. You cannot do better than write to her on the subject. Her pupils have done well.

*Pneumatic Tyres.*

(213) Please tell me where I can get pneumatic tyres for an invalid couch, and if they could be fitted to the existing wheels?—*S. P., Switzerland.*

Write to Messrs. Leveson and Sons, 90 and 92, New Oxford Street, W.C., sending particulars of the size and number of the wheels, and the exact measurement of the axle. Pneumatic tyres could not be fitted to ordinary wheels, but new wheels can be sent out to you from England.

*Weekly Dietary.*

(214) What should you consider a fair and reasonable sum for the weekly dietary of an adult patient in an isolation hospital?—*Sister Sarah.*

In hospitals under the Metropolitan Asylums Board 10¼d. is allowed per day for the maintenance and clothing of each patient. At the small-pox hospital ships 1s. 1½d.

*Fever Nursing.*

(215) Can I get into an isolation hospital for training, and how long a time must one give to it? I am just over 40, so suppose I am too old to enter a hospital except as a paying probationer.—*A Would-be Nurse.*

The London fever hospitals do not, as a rule, take probationers at all, the assistant nurses must have had some general training. At your age we fear you will find it difficult to get what you want, but you would probably do well to advertise in our columns, and you might also apply direct to the matrons of isolation hospitals. You will find a list of these, London and provincial, in Burdett's "Hospitals and Charities" (Scientific Press, 28 and 29, Southampton Street, Strand, London, W.C.) We were interested in your letter, and are very glad to be of any assistance to you.

*Preliminary Examination.*

(216) I have applied at St. Bartholomew's for a vacancy as probationer, and I find there is an examination to pass before entering the hospital. Can you please tell me whether the hospital prepares candidates for this examination, or must one be prepared beforehand?—*Reader.*

You should have asked these questions when you applied at the hospital, when doubtless you would have received all particulars. You had better write to Miss Stewart (the matron), and ask for particulars of the examination questions, and if any special books are recommended for the study of would-be probationers.

*Training.*

(217) I am twenty years old, and well educated. How can I obtain a post as probationer in a hospital, taking a small salary if possible?—*Would-be Pro.*

Read "How to Become a Nurse" (Scientific Press, 28 and 29, Southampton Street, Strand, London, W.C.), and apply at the various hospitals you will find there mentioned where probationers are eligible at your age.

*Bicycles for District Nurses.*

(218) Sister Edith will be very grateful if superintendents and district nurses who use bicycles will kindly tell her if they find it a real help and saving of time. If so, she hopes to persuade her committee to get one for her nurses. Letters directed to Sister Edith, care of the Editor, will be forwarded to her.

*Nurses for Crete.*

(219) I see in this week's HOSPITAL that several nurses have sailed for Crete. Will you please give me some information on the subject, and tell me if any more nurses are required, as I should like to volunteer? I have a three years' certificate.—*Trained Nurse.*

We understand that Mrs. Ormiston Chant, Lady Henry Somerset, and Mrs. Bedford Fenwick were responsible for the expedition to Crete, and for the collection of the necessary funds. It was purely a private enterprise, and you must apply to one of the ladies mentioned for information. See our rules respecting answering letters by post.

"Nurse" is reminded that name and address must accompany all letters requiring an answer in this column.

## Figures and Tables

**Figure f1:**
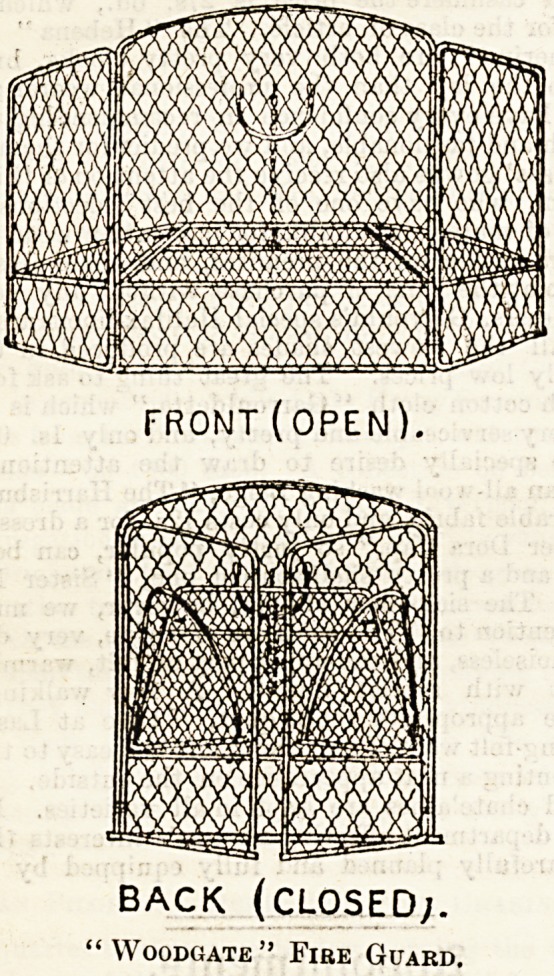


**Figure f2:**
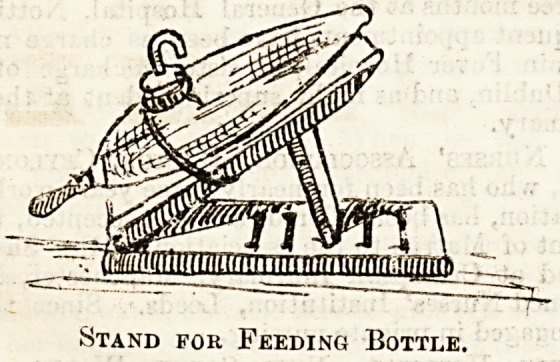


**Figure f3:**